# Porting and using PanGIA for Cytoscape 3: challenges and solutions

**DOI:** 10.12688/f1000research.4546.1

**Published:** 2014-07-01

**Authors:** David Welker, Barry Demchak

**Affiliations:** 1 University of California, San Diego, La Jolla, CA 92093, USA

## Abstract

Much of the biologically significant functionality in Cytoscape is contained within third-party add-ons, called plugins in Cytoscape 2 and apps in Cytoscape 3. In the transition from Cytoscape 2 to Cystoscape 3, some of the underlying assumptions upon which plugins relied changed, requiring a significant porting effort for plugins to work as Cytoscape 3 apps. PanGIA is a Cytoscape add-on (http://apps.cytoscape.org/apps/pangia) designed to analyze and visualize genetic interaction data in light of physical interaction data. In order to convert the PanGIA plugin to an app, various challenges, including those related to a transformed data model, concurrency, and randomization had to be overcome. In the process, the ability to control randomization was added to the GUI, a feature which was not only integral to the porting process, but which also ensures more easily reproducible scientific analysis for PanGIA users. Most authors of Cytoscape 2 plugins will face similar challenges porting their software to work with Cytoscape 3, and this paper gives details of how the PanGIA port addressed them.

## Introduction

PanGIA (which stands for Physical and Genetic Interaction Alignment) is a Cytoscape plugin originally developed for Cytoscape 2, and which has now been released for Cytoscape 3. The purpose of PanGIA is to help determine the functional significance of genetic interaction data, which is being increasingly uncovered by a plethora of modern high-throughput technologies. It identifies genetic interactions in the context of physical protein-protein interactions by identifying both “within-cluster” physical and genetic interactions, which may signify either functional protein complexes or signaling pathways, and “between-cluster” genetic interactions, which often identify synergistic or compensatory relationships between clusters Srivas *et al.* ^1^. In order to ensure the ability to perform such analysis was widely available to scientists, PanGIA was originally released as a novel open source software tool that worked as a plug-in for Cytoscape 2. Due to sufficient demand by researchers, we have ported PanGIA as an app (the new term for plugin) for Cytoscape 3.

The challenges we encountered in the porting process and our solutions to them are the primary focus of this paper. As a secondary focus, this paper provides a brief overview of how to use PanGIA and the results it produces. Readers of this paper who are not app developers interested in porting their plugin from Cytoscape 2 to Cytoscape 3 may want to skip the “Porting PanGIA” section of the paper. The remainder of this paper consists of three primary parts: a porting section that highlights the main technical challenges we encountered in porting PanGIA, a brief overview of how to run PanGIA, and a final section that provides a brief overview of the meaning of PanGIA’s outputs.

## Porting PanGIA

To port a Cytoscape 2 plugin to a Cytoscape 3 app, a good understanding of Java and Swing is necessary. Often, deficiencies in knowledge of these technologies can be overcome by referencing Oracle’s Java Tutorials ( http://docs.oracle.com/javase/tutorial/). The rest of this section assumes basic familiarity with Java and Swing. Additionally, for those looking for basic information on how to port an app to Cytoscape 3, a good place to start is the Cytoscape Plugin Porting Guide ( http://wiki.cytoscape.org/Cytoscape_3/AppDeveloper/PluginPortingGuide).

In our port of PanGIA, we encountered three main challenges. First, the manner in which data is stored concerning nodes, edges, and networks is significantly different in Cytoscape 2 versus Cytoscape 3, and it was rather time-consuming to perform the conversion. Second, it was necessary to explicitly ensure that the Swing dialog messages that PanGIA displayed to the users were run on the EDT in order to avoid deadlocks (Whereas in Cytoscape 2, tasks often run on Java’s event dispatch thread (“EDT”) by default, in Cytoscape 3, they do not.) Finally, since PanGIA involves heuristic algorithms with a random element, it was necessary to devise a means to control such randomization so that the same results on the same data could be achieved in both Cytoscape 2 and Cytoscape 3 in order to confirm correctness. Besides these three main challenges, there were numerous smaller challenges. For example, we had to create a CyActivator to register objects relating to the new Cytoscape OSGi framework, and to create context menu items.

A useful reference for performing OSGi-related developer work is the Cytoscape Samples Repository ( https://github.com/cytoscape/cytoscape-samples), since it contains many hands-on examples of such work (such as all the steps for registering context menu items) that are not documented as well elsewhere. The Cytoscape 3 App Cookbook ( http://wiki.cytoscape.org/Cytoscape_3/AppDeveloper/Cytoscape_3_App_Cookbook) is also an invaluable resource that ought to be consulted regularly by developers looking to port their plugins.

The remainder of this section briefly describes each of the three main challenges to porting that we encountered in turn.

### Transformed data model

The most time-consuming aspect of porting PanGIA was adapting to the numerous changes in the manner in which data is stored in Cytoscape 3 compared to Cytoscape 2. While this type of work is usually straight-forward, depending on the plugin, changes are likely to be extensive since most plugins interact with the underlying network data model extensively.

One issue with the Cytoscape 3 data model is likely to be counterintuitive to novice plugin porters in particular. In general, when adding data to a node table or edge table, one usually must first add an appropriate column to the table object. Based on this, one might intuitively think it is most common to put the data directly into the table object as well. But such an inference would be incorrect; it is much more common and convenient to add the data using the network object. This is done because before new data can be added to the table, a new row must be created. But to create a new row on the table object, one must call an appropriate method on the network object, not the table object. After doing this, although it is possible to add the data using the table object directly, it is much more common to use a convenience method on the network object to add the data to the table indirectly. The code snippet below is an example of working with the data model in Cytoscape 3 in the context of a node table:



                        public void 
                        method(CyNetworkFactory factory)

                        {
 
                        //Create a new network and get node table.
 
                        CyNetwork network = factory.createNetwork();
 
                        CyTable table =                
     network.getDefaultNodeTable();
     
 
                        //Create the column in the table object.
 
                        table.createColumn(
                        "MyColumn"
                        , String.
                        class
                        ,
     
                        false
                        );
     
 
                        //When a new node is created with addNode(),
 //a new row is also created in the table.
 
                        CyNode node = network.addNode();
 
 
                        //Most commonly, data for the new node is
 //added using the network object, not the
 //table object.
 
                        network.getRow(node).set(
                        "MyColumn"
                        , 
                        "New
     Data"
                        );
}
                    


This contrasts with Cytoscape 2, where data was usually added to CyAttributes (the approximate equivalent of CyTable) directly, rather than most commonly going through another object. The code snippet below shows the typical case of data being added directly to the CyAttributes object directly:



                        public void 
                        addDataToTable(CyNode node,
    CyAttributes attributes, Integer data)
{
  
                        //In Cytoscape 2, data is added directly to
  //the attributes object.
  
                        attributes.set(node.getIdentifier(),
     
                        "MyAttribute"
                        , data);
}
                    


Using one object to create a column and then another to create a row and add data is just an example of one of the more tricky transformation issue that developers are likely to face. In general, Cytoscape’s powerful and flexible data model is not always intuitive at first, but the process becomes easier with time. To port a plugin to Cytoscape 3, the first task is usually understanding the Cytoscape 3 data model and performing the conversion.

### Concurrency

Part of the standard mechanism in Java for handling concurrency is to ensure that nearly all invocations of Swing components occur on the EDT. (See The Java Tutorial: The Event Dispatch Thread at http://docs.oracle.com/javase/tutorial/uiswing/concurrency/dispatch.html). In Cytoscape 2, tasks were invoked on the current thread, which in many cases was the EDT. This allowed some plugins to implicitly rely on the fact that they were probably being executed on the EDT. However, in Cytoscape 3, every task is spawned on a new thread, which means that when work is done in a task, by default, it does not run on the EDT. Because PanGIA relied on the old behavior, it was necessary to explicitly make sure that any messages to be displayed to the user using Swing dialogs were run on the EDT. This was typically done using the SwingUtilities.invokeLater method using an anonymous inner class. Code that needs to be run on the EDT, such as any invocations of Swing components, is simply executed in the run method of an anonymous Runnable object which is passed as a parameter into the invokeLater method. The code snippet below illustrates:



                        SwingUtilities.invokeLater( 
                        new 
                        Runnable()
{
  @Override
  
                        public void run()
  {  
   
                        //Code which must be executed
   //on the EDT goes here.
  
                        } 

                        }
                    


Since this is an anonymous inner class, if it uses any data from the method or class it is embedded in, that data must be declared final. In the instances where this is a problem, the goal of data access can usually be accomplished by declaring a new final variable and assigning the non-final data to that. Then, it is the new final variable, not the original data, that would be accessed in the run method. Finally, although it is permissible to do non-Swing work on the EDT, such work ought to be minimized, as this has the potential to cause the GUI to hang because such work is given a higher priority than repainting the GUI.

### Randomization

In PanGIA, the algorithms used to determine the clusters of genes (or modules, as PanGIA refers to them) use a random number generator that influences both the assignment of genes to modules and the number of modules produced. Therefore, this random number generator made it very difficult to determine the correctness of the port of PanGIA from Cytoscape 2 to Cytoscape 3, since different clustering arrangements occurred simply as a matter of chance. Fortunately, the random numbers in Java and other programming languages are not truly random, but rather pseudorandom. (See the API for the Random class at http://docs.oracle.com/javase/7/docs/api/java/util/Random.html). This means that, unlike with truly random numbers, we can eliminate unpredictability and instead create repeatability. When unpredictability isn’t desired, it is possible to take control of the random number generation process by setting an initial value (called a seed) for the Random class explicitly. For our port of PanGIA, we took advantage of this to prevent unpredictability in order to verify that the same results were produced by both versions of the PanGIA add-on.

PanGIA for Cytoscape 2 had multiple locations where instances of the Random class were instantiated in a manner that did not control randomness. In order to control randomness for these instances, we created a single seed variable and added the ability to set this seed variable from the GUI. We then changed each of these locations so that they constructed Random objects using this seed variable. Upon choosing any arbitrary value for the seed, we saw that results were now reproducible, just so long as the same arbitrary value was consistently chosen. In order to compare results across Cytoscape 2 and Cytoscape 3 to ensure correctness, we also added the same seed functionality to the PanGIA plugin for Cytoscape 2 and saw that when the same data was used in both versions, the same outputs were produced. This sort of black box testing was only possible when additional functionality was added to control randomization.

## Running PanGIA

Running PanGIA is fairly straightforward. First, one must load the physical and genetic network. Then one must set appropriate parameters using PanGIA’s graphical user interface (GUI). Finally, one hits the Search button and PanGIA runs for an amount of time that depends on the size of the physical and genetic networks and outputs the results. The meaning of the input parameters and advice on setting them are well explained in Srivas *et al.* ^1^. With only small deviations, the very detailed steps in that paper are still applicable and we recommend that users who need a detailed understanding of how to run PanGIA continue to reference that paper as their primary guide to setting input parameters.

However, there is one new feature that PanGIA users will find interesting which does not exist in the version of PanGIA released to work with Cytoscape 2, and that is the ability to control randomization by setting the seed parameter in the GUI. Users should know that PanGIA’s core algorithms are non-deterministic because of their dependence on random number generation. This means that with the previous version of PanGIA, a user’s results may not be perfectly reproducible in the future. But if the user needs reproducibility for their research, with PanGIA for Cytoscape 3, it is now possible. In the new PanGIA GUI, there is a new parameter that can be set called Seed. The user simply sets this to any integer of their choosing (in the range of −2 ^63^ to 2 ^63^−1). As long as the same number is used each time PanGIA is run on the same data set with all the other parameters held constant, the same results will be output.

## Visualizing and analyzing results

**Figure 1.**
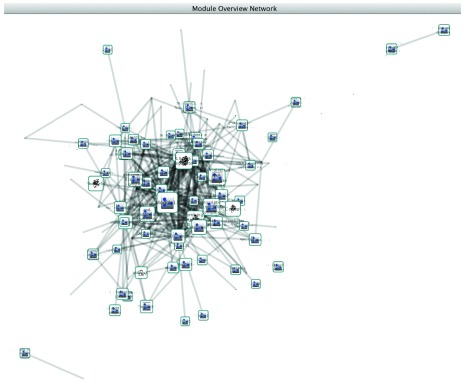
The module overview network.

**Figure 2.**
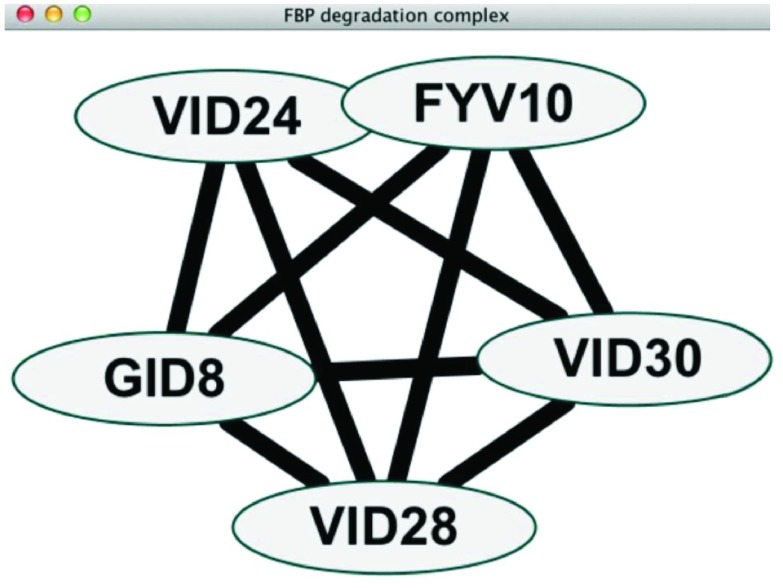
Within-cluster interactions.

**Figure 3.**
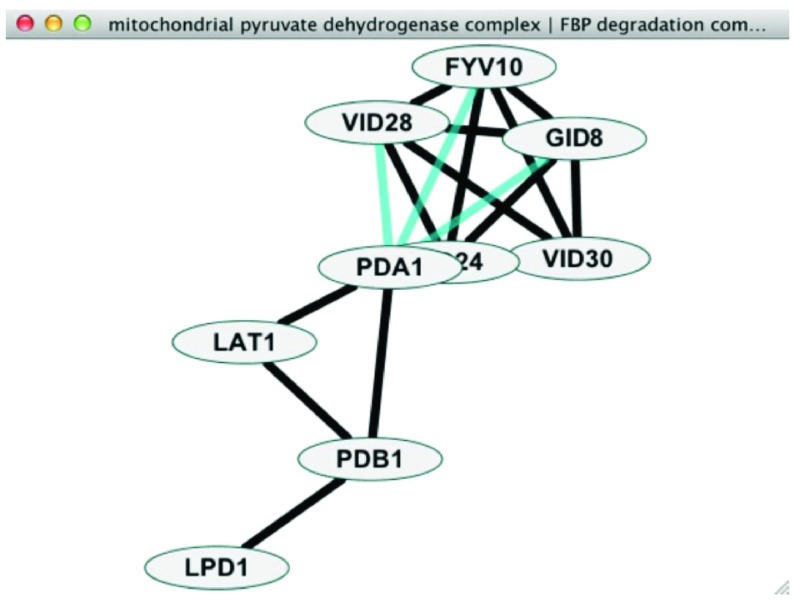
Between-cluster interactions (in blue).

After running PanGIA, a large number of networks will result. One of these networks will be the overview network while all the others will consist of modules of genes with significant physical protein-protein interactions and associated genetic interactions. These networks will allow the user to visualize the overview network ( Figure 1), within-cluster interactions ( Figure 2), and between-cluster interactions ( Figure 3). The remainder of this section will consider each of these in turn.

### The overview network

The overview network consists of a higher level view of the results, where the nodes are the modules that PanGIA found and the edges represent genetic interactions that exist between those modules. From the overview network, the user can see between-cluster genetic interaction generally, but not the specific genes in each module that are involved. Thus, the overview network is good for a high-level overview of between-cluster genetic interactions between modules as well as a sense of how many and which modules were discovered.

### Within-cluster interactions

Within-cluster interactions can be examined by selecting a node within the overview network and right-clicking and choosing Apps | PanGIA | Create Detailed View. Black edges represent physical interactions, light yellow edges represent positive genetic interactions, and light blue edges represent negative genetic interactions.

### Between-cluster interactions

To visualize between-cluster interactions, the user selects two or more nodes in the overview network and right-clicks to bring up the context menu and chooses Apps | PanGIA | Create Detailed View. For the selected nodes, the user will still see within-cluster physical and genetic interactions. In addition, the user will see the genetic interactions that connect the two or more modules and the specific genes involved.

## Conclusions

In this paper, we have explored some of the challenges we faced when porting PanGIA which may be applicable to developers looking to port other plugins. We also briefly explained how running PanGIA may be affected by the new random seed feature and briefly examined the outputs from running PanGIA.

### Software availability

Software available from http://apps.cytoscape.org/apps/pangia

Latest source code https://github.com/idekerlab/pangia3/

Source code as at the time of publication https://github.com/F1000Research/pangia3/releases/tag/V1.0

Archived source code as at the time of publication http://www.dx.doi.org/105281/zenodo.10459 ^2^

License Lesser GNU Public License v2.1

## Supplementary data

The data necessary to reproduce the figures used in this paper were taken from the website for Srivas *et al.* ^1^ ( http://prosecco.ucsd.edu/PanGIA/) (available as physical_network.txt, genetic_network.txt, and annotations.txt). Please load the physical and genetic networks before the annotations.
