# Supplementary material for: Porting and using PanGIA for Cytoscape 3: challenges and solutions
Source: F1000Res. 2014 Jul 1;3:150. [Version 1] doi: 10.12688/f1000research.4546.1 (PMC4103495; doi:10.12688/f1000research.4546.1)
Supplement: Peer review history

# Peer review: Referee response for version 1

**DOI:** 10.5256/f1000research.4863.r5296

**Published:** 2014-07-14

**Authors:** Alfredo Pulvirenti

**Affiliations:** 1 Department of Mathematics and Computer Science, University of Catania, Catania, Italy

In this paper the authors describe their experience in the porting of PanGIA to Cytoscape 3. The paper aims to: (i) point out to the developers the main tricks and challenges to perform the porting from Cytoscape 2 to 3; (ii) describe the new feature in the last PanGIA plugin.

The paper is interesting and it certainly could result in being useful to developers and users.

**Minor issue:**

The authors emphasize the novel feature allowing reproducing the results by controlling the seed of the random number generator. This is certainly an important point, however I would reduce the paragraph since it describes really basic programming stuff. On the other hand, I would stress the point that such a “seed control” should be always available in all the systems using randomization. Furthermore, in the “running PanGIA paragraph” the description of the “seed setting” is quite repetitive and should be also reduced.

I have read this submission. I believe that I have an appropriate level of expertise to confirm that it is of an acceptable scientific standard.

# Peer review: Referee response for version 1

**DOI:** 10.5256/f1000research.4863.r5294

**Published:** 2014-07-14

**Authors:** Brian Kidd

**Affiliations:** 1 Genetics and Genomic Sciences, Mount Sinai Hospital, Toronto, ON, Canada

This article provides useful technical advice for Cytoscape app developers looking to port plugins from version 2 to apps in version 3. The technical advice is provided in the context of a specific application PanGIA, which helps determine the functional significance of genetic interaction data. The case study of PanGIA describes:

1. How to deal with the data model and GUI event handling changes for developing software within the Cytoscape version 3 framework.
2. What upgrades have been introduced in the port from v2 to v3, and some expected results when running the software.

Overall the authors do a nice job of giving development advice and technical details for creating a Cytoscape 3 app if one is familiar with creating plugins for Cytoscape 2. In addition, the authors describe the new functionality that was added to the PanGIA app (random seed for obtaining reproducible results from the heuristic algorithm) and offer a small tutorial for anyone wanting to use the app for their research.

This article is clearly written and provides a helpful resource for Cytoscape developers as well as end-users.

I have read this submission. I believe that I have an appropriate level of expertise to confirm that it is of an acceptable scientific standard.
